# Somatic Mutations and Autoimmunity

**DOI:** 10.3390/cells10082056

**Published:** 2021-08-11

**Authors:** Maha Alriyami, Constantin Polychronakos

**Affiliations:** 1 Department of Biochemistry, College of Medicine and Health Sciences, Sultan Qaboos University, Muscat 123, Oman; mahazr@squ.edu.om; 2 The Endocrine Genetics Laboratory, Child Health and Human Development Program, Department of Pediatrics, McGill University Health Centre Research Institute, Montreal, QC H4A 3J1, Canada

**Keywords:** somatic mutations, autoimmunity, lymphocytes

## Abstract

Autoimmune diseases are among the most common chronic illness caused by a dysregulated immune response against self-antigens. Close to 5% of the general population in Western countries develops some form of autoimmunity, yet its underlying causes, although intensively studied, are still not fully known, and no curative therapies exist. It is well established that autoimmune diseases have common mechanisms and are caused by both genetic and non-genetic risk factors. One novel risk factor that can contribute to autoimmunity is somatic mutations, in a role parallel to their role in cancer. Somatic mutations are stochastic, *de novo*, non-inherited mutations. In this hypothesis, the persistent proliferation of self-reactive lymphocytes (that is usually hindered by a series of checkpoints) is permitted, due to somatic mutations in these expanding cells, allowing them to bypass multiple regulatory checkpoints, causing autoimmunity. This novel concept of the contribution of these mutations in non-malignant diseases has recently started to be explored. It proposes a novel paradigm for autoimmunity etiology and could be the missing piece of the autoimmunity puzzle.

## 1. Introduction

Autoimmune diseases are among the most prevalent chronic illness caused by a dysregulated inflammatory response against self-antigens (such as the pancreatic insulin-producing beta cells in type 1 diabetes (T1D), the myelin sheath in multiple sclerosis (MS), and the chromatin in systemic lupus erythematosus (SLE)). Close to 5% of the general population develops some form of autoimmunity, and the complexity of the geoepidemiology of these diseases rises when considering some aspects, such as age, gender, ethnicity, and other demographic features [1].

Immunologists and geneticists have been joining efforts to decode the immune system and determine the genetic and cellular fundamentals of its functional and self-tolerance pathways, which, when disturbed, cause autoimmunity. Despite the rigorousness and effectiveness of the central and peripheral tolerance mechanisms, a minute number of potentially self-reactive lymphocytes escape into the periphery. However, their mere existence is not sufficient to cause disease; autoimmunity can be physiological, a temporary state with no clinical symptoms or pathology [2]. The underlying causes of autoimmune diseases, despite the intensive research, are still elusive, and curative therapies are nonexistent. Contemporary studies revealed that the break in the immune tolerance causing various forms of autoimmunity has common cause mechanisms, and the development of pathological autoimmunity necessitates both genetic and non-genetic risk factors [2].

In today’s genomic era, the genetic component of the autoimmunity equation is well recognized and established, with the chief risk loci having been identified, contrasting with the environmental component that remains unclear. However, the current knowledge does not explain the discordance in autoimmunity observed in monozygotic twins living in a shared environment (at least for early-onset diseases, such as T1D) and in certain inbred animal models housed in a stable, constant, and controlled environment and, therefore, identical for both genome and environment. Moreover, it does not explain the delayed stochastic penetrance of autoimmunity. Stochastic events may explain these observations of discordance.

One potential stochastic event is the existence of somatic mutations in the expanding autoimmune cells as a common pathogenetic mechanism for all forms of autoimmunity. In this hypothesis, somatic mutations, occurring as self-reactive lymphocytes that proliferate after contact with antigen, might interfere with one component of the series of self-tolerance checkpoints designed to arrest their persistent proliferation [3]. Proliferation creates additional opportunities for somatic events that might allow these lineages to bypass multiple regulatory checkpoints, causing autoimmunity. This somatic mutation hypothesis draws an overlap in the underlying molecular mechanisms of the pathogenicity of autoimmunity and cancer [3]. Moreover, it is, perhaps, the missing piece of the autoimmunity puzzle, and the current genomic advances make testing this hypothesis possible, offering novel avenues for research in the field of autoimmunity and its tailored, personalized treatments.

Here, we reviewed recent studies examining this hypothesis. We began by discussing the pathogenesis of autoimmunity. We then described the somatic mutation hypothesis and the evidence supporting it. The current research supporting this hypothesis is then discussed. We then looked at how this hypothesis could be harnessed for personalized medicine. Finally, we outlined the future directions for the field.

## 2. The Pathogenesis of Autoimmune Diseases

The central role of the various pleiotropic immune cells is to safeguard the host from infectious agents. Failure to do so results in one of two pathological manifestations: first, immunodeficiency syndromes, in which one or more modules of the immune system’s capability of reacting protectively to pathogens is compromised (or entirely absent) or, alternatively, autoimmunity, in which the immune system launches an immune response against its own, due to failure in identifying self-cells [2].

The immune system must be able to differentiate self-antigens from alien antigens, in order to function properly. This necessitates maintaining a delicate balance between host defense and self-tolerance. To equip the immune system’s lymphocytes with various antigen-detecting receptors, a number of physiological mechanisms of V(D)J recombination and somatic mutation exist [4]. Lymphocytes have the ability to turn on robust and powerful cell growth and survival pathways when encountering an antigen. This is due to the membrane-spanning immunoglobulin isoforms: the T cell receptors (TCRs) expressed on T lymphocytes and the B cell receptors (BCRs) expressed on B cells.

Tolerance mechanisms in central lymphoid organs play a fundamental role in shaping immune system homeostasis; these include positive and negative selection in the thymus. This is enhanced by the capacity of the thymic medullary epithelial cells to express a diverse range of ordinarily tissue-specific antigens. The first demonstration of a tissue-specific antigen expression in the thymus was the thymic expression of insulin in low levels, correlating with a variable number of tandem repeats (VNTR) polymorphism that strongly protects from T1D [5]. Beyond the thymus, tolerance mechanisms in the periphery include mechanisms of clonal deletion, ignorance, anergy, and immune regulation. Immunological self-tolerance mechanisms are reviewed in detail elsewhere [4,6].

The genetic component of autoimmunity (with the exception of rare Mendelian autoimmune diseases) results, substantially, from common variants of the major histocompatibility complex (MHC) Class II molecules that modulate the efficiency of antigen presentation [7]. However, the rest of the genetic component comes from the additive effects of multiple genetic loci cooperating in causing autoimmunity, each with a little or no effect alone [7].

**Figure 1:**
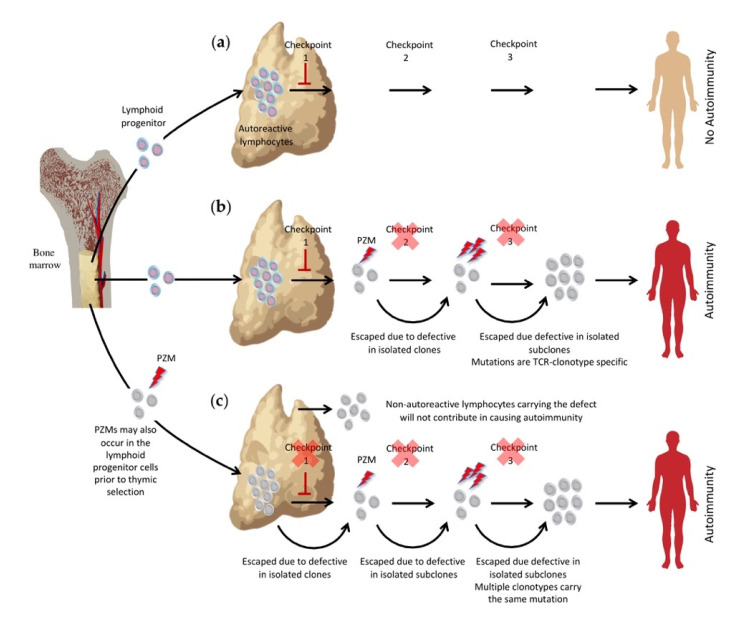
The somatic mutation hypothesis. Many lymphocytes clones, generated in the bone marrow, carry autoreactive receptors. (**a**) Normally, the generated autoreactive lymphocyte clones are eliminated by multiple growths and survival checkpoints, preventing autoimmunity; (**b**) post-thymic somatic mutations (lightning bolt) in a self-reactive lymphocyte clone (mutated cells in grey) might allow its bypass of these checkpoints and the potential accumulation of further mutations disrupting other checkpoints, causing the onset of stochastic autoimmunity; (**c**) somatic mutation events might take place pre-thymic in bone marrow progenitor cells and not in the antigen-specific lineage. Here, lymphocytes with no autoreactive receptors will not contribute in causing autoimmunity.

The development of most autoimmunity takes a considerably prolonged time, both in human and experimental animals. This latent period is required for the complete evasion of the tolerance checkpoints, by specific autoreactive T cells. An example of this latency can be seen with mutations of the autoimmune-regulator (AIRE) that controls the transcription of organ-specific genes in the thymic epithelial cells, allowing their expression of a diverse range of ordinarily tissue-specific antigens [8]. Inherited *AIRE* mutations affect the thymic negative selection, resulting in a multi-organ disease autoimmune polyendocrinopathy syndrome type 1 (APS1). AIRE deficiency remains clinically silent for several years before manifesting in a stepwise, stochastic manner [9]. This suggests that the self-reactive clones that leak through thymic deletion must escape several independent checkpoints before causing autoimmunity [3]. These checkpoints could be inactivated by successive somatic mutations as the cell lineage proliferates, which is the concept of the somatic mutations in autoimmunity hypothesis (Figure 1) [3].

## 3. The Somatic Mutation Hypothesis

### 3.1. The History of the Somatic Mutation Hypothesis

Throughout the early twentieth century, a misconception prevailed that the healthy body is incapable of threatening itself by producing “forbidden clones”, which are immune cells capable of recognizing and mounting an immune response against self-antigens, a theory described as “The horror autotoxicus” by the pioneer in the elucidation of humoral immunity, Paul Ehrlich [10,11]. Around the same time, Metalnikoff accepted the possibility of autoimmunity by showing, in animal studies, the generation of antibodies attacking their own sperm [11,12]. Paul Ehrlich maintained his position, explaining that these are not considered “autocytotoxins”; hence, the attack of sperms is not taking place in their usual vivo site [13], which clarifies the true meaning of horror autotoxicus theory: that antibodies against self-antigens can be generated, but are inhabited by some mechanisms [11].

In 1959, Sir Frank Macfarlane Burnet proposed, in his ground-breaking “the clonal selection theory of acquired immunity”, his ‘forbidden clone hypothesis’, stating that immunological un-reacting to self-antigens, via clonal deletion, is a feature adopted during development that, works in safeguarding against “horror autotoxicus” and that autoimmune disease develops as a consequence of persisting self-reactive clones. Burnet postulated that somatic mutation, early in lymphoid differentiation, provides stochastic mechanisms disrupting tolerance and resulting in the formation of the forbidden clones. [14,15,16]. In 2007, the hypothesis was proposed again by Christopher Goodnow in his *Cell* review, pointing out that the somatic mutation may grant the self-reactive lymphocytes a growth advantage, allowing for a higher potential for selection and accumulation of further mutations in a single clone. Moreover, genetic defects in one cell can collaborate with defects in another cell to stimulate the override of growth-regulatory checkpoints [3]. Goodnow emphasized the potential role of somatic mutations in the pathogenesis of autoimmune diseases in a paradigm similar to that in lymphoid cancer, combining perceptions from the latter to augment the understanding of autoimmunity. Goodnow points out that when an antigen is encountered, the TCR and BCR, activates growth and survival pathways such as: the transcription factor Kappa B (NF-_k_B), phoosphatidylinositold-3-kinase (PI3K), Ras, MYC, BCL-2, and BCL-XL. In lymphoid cancers, these same cell growth and survival pathways are disrupted and have uncontrolled activity; for example: in several human lymphomas, chromosomal translocations that activate MYC and BCL-2 are observed [3].

The somatic mutation hypothesis suggests a range of effects on the immune system by affecting the molecular signaling pathways promoting proinflammatory signaling and cell survival of immune cells, disrupting the immune balance required for self-tolerance.

### 3.2. Somatic Mutations

Although DNA replication is high-precision machinery, errors sporadically occur, creating mutations. This creates continuous modifications in the genome, enabling evolving and adaptation. Mutations (copy-number [17] and point mutations) are classified as germinal/meiotic mutations or somatic mutations. Unlike germinal mutations, somatic mutations are genomic alterations that are not transmitted to offspring. They occur at any point during development in which a mutation, occurring in a progenitor cell, will be passed to all descended daughter cells and multiple tissue types. Moreover, the earlier the time point that the somatic mutations occur during embryogenesis, the greater the number of progeny cell clones carrying it [18,19].

When a somatic mutation is confined to a single post-mitotic cell in a fully developed organism, their effect can be insignificant, whereas, when they occur in cell types with a proliferative property, they can result in mutant clones [20]. The contribution of somatic mutations to tumorigenesis, in which they affect tumor suppressor genes and oncogenes, is well-acknowledged and established, causing pathological phenotypes and an escape from proliferation controls [21,22]. In addition, it facilitated therapeutic advances by identifying drug targets that are essential in disease causative [22]. One example here is Gleevec, which is a drug that acts as a selective inhibitor of the chronic myeloid leukemia (CML) causal fusion transcript BCR-ABL tyrosine kinase [23].

### 3.3. Somatic Mutations in Non-Malignant Diseases

Copy-number mosaicism, detectable in whole-blood, is common but only a minute fraction of carriers develops leukemia [24], pointing to a broad gray zone for potential involvement in non-malignant blood cell diseases.

In a manner parallel to lymphoid cancer, autoimmune diseases are non-malignant diseases, yet they are caused by the proliferation of lymphocyte clones originating from a small number of autoreactive ones that have escaped natural tolerance mechanisms. In addition to their shared pathogenesis with cancer (proliferation and apoptosis resistance), autoimmunity requires more functional steps. Natural tolerance entails several rigorous checkpoint mechanisms that prevent the expansion and activation of autoreactive lineages. Failure of this multistep process results in autoimmunity manifestations over several years. Contemporary research has confirmed that a substantial fraction of newly generated immune cells has receptors capable of detecting and binding to self-antigens [25]. This phenomenon is a by-product of the purposefully random V(D)J recombination mechanism, which generates T-cell and B-cell receptors. Approximately 20–50% of TCRs and BCRs generated via the V(D)J recombination process identify and bind to self-antigens with a potentially high-risk affinity [26,27,28]. Normally, most of these are eliminated by apoptosis in the thymus by negative selection through BIM-induced apoptosis [29] or FAS/CD95 receptor pathway [30,31]. However, some escape to the periphery, where they are dealt with by peripheral tolerance. Several genes and cellular mechanisms have been identified to be involved in immunological self-tolerance, preventing autoreactive cells from exerting any destructive action. When impaired, the self-reactive cells and autoantibodies react against its self-antigens, causing their destruction and therefore, autoimmunity. In the absence of genetic variance (inbred strains and twins), and despite substantial evidence to the contrary from animal models raised in germ/antigen-free environment, the variability of the latent phase and stochastic onset of autoimmune illness are frequently attributed to an unknown environmental trigger. In his review, Goodnow (2007) pointed that autoimmunity shares this latent, sporadic occurrence with lymphoma. However, in cancer, this has been attributed to the necessary accumulation of somatic mutations for the mutate clones to bypass growths and regulatory checkpoints. For example, inherited mutations in the tumor suppressor gene *p53* have a delayed stochastic penetrance due to the time required for the accumulation of multiple somatic mutations in a single clone to sidestep other regulator layers [32]. Goodnow pointed that similarly, there are multiple control checkpoints that reduce or override TCR and BCR receptor signaling, preventing immune cells from proliferating [3]. These checkpoints are controlled by genes known to suppress lymphoid cancers. The disruption of these genes has been associated with autoimmunity and lymphoid cancers, such as BIM deficiency [33] and *FAS* mutations [34,35]. This mechanistic overlap between autoimmunity and cancer supports the somatic mutations hypothesis, proposing a potentially significant stochastic role for somatic mutations in the pathogenesis of autoimmunity.

### 3.4. Frequency of Somatic Mutations

The plausibility of the somatic mutation hypothesis in autoimmunity causation depends on their frequency, specifically in autoimmune-relevant hematopoietic lineages [36]. The attempts to quantify the rate of somatic mutations started by evaluating small genomic loci such as *PIG-A* and *HPRT* genes [37,38].

For non-hematopoietic cells, recent single-cell sequencing studies revealed that somatic mutations commonly occur and accumulate in normal cells, with a frequency ranging from 3.5 × 10^−9^ mutations/bp/division (in the small intestine) to 1.57 × 10^−7^ mutations/bp/division (in the skin) [19,39,40,41,42,43,44,45]. The estimated frequency of somatic mutations in non-hematopoietic cells increases steadily with age, with an approximate rate of 40 novel mutations per year [41].

In hematopoietic cells, somatic mutations originating in hematopoietic stem cells and progenitor cells can create a mosaic state, occasionally detectable in the whole peripheral blood of healthy individuals [24,46,47,48,49,50,51]. Somatic mutations may occur in healthy individuals, in genes with malignancy implications, such as *DNMT3A, TET2*, and *ASXL1* making them predisposed to hematological malignancies [47,52]. The first in vitro estimates of the mutation rate in myeloid cells were ~10^−6^ mutations per gene/division [53]. Lymphocytes undergo somatic mutations as part of their normal maturation processes, such as the V(D)J recombination process, somatic hypermutation (specialized B-cell maturation mechanism), and isotype switching. However, these are error-prone mechanisms and may trigger oncogenes and deactivate tumor-suppressor mechanisms resulting in lymphomas [54].

SNP-array data revealed megabase-range mosaic somatic aberrations in about 3.4% of healthy individuals with a clonal frequency of (5–10%) [24,50]. The detectable mosaicism increases with age, suggesting that their escalation to detectable levels results from a proliferation/survival advantage [24,50,51]. A monozygotic twin study aimed to identify SNP-array discrepancies from whole blood and about ~130 point- somatic mutation per individual was identified [55,56]. The frequent occurrences of somatic mutations in hematopoietic cells demonstrated the case of Paroxysmal nocturnal hemoglobinuria (PNH). PNH is an autoimmune disease characterized by hemolytic anemia and caused by a mutation in a hematopoietic stem cell in the *PIG-A* gene. This mutation disrupts the attachment of the complement inhibitory receptors (CD48, CD55, and CD59) to the cell surface. The frequency of somatic mutations in *PIG-A* was estimated as 22 affected blood cells per million [57], with an emergence of a new deleterious mutation in every million cell divisions [37].

Since the peripheral whole-blood is a heterogeneous mixture, the frequency of somatic mutations in it is undoubtedly underestimated, and their mosaicism level is too low to be detectable by conventional approaches [51]. However, due to the logarithmic expansion of the autoantigen-specific lineages, mosaicism can be estimated to be much greater; yet, its detectability would be low without isolating the specific lineages from peripheral whole-blood. It is, therefore, when considering the somatic mutations rate of ~10^−6^ mutations per gene/division [53], likely that lymphocytes carry an inherited deleterious mutation in a heterozygous state. In a manner similar to cancer, the unmutated functional copy will be at risk of disruption due to a somatic mutation (second hit) resulting in the sporadic manifestation of autoimmunity [3]. Alternatively, haploinsufficiency or gain of an abnormal function, might promote autoimmunity, even in the heterozygous state.

### 3.5. Epidemiological Evidence for the Somatic Mutation Hypothesis

The overlapping pathogenesis of autoimmunity and lymphoma, observed in epidemiological studies, points to somatic mutations as a crucial stochastic factor in the pathogenesis of autoimmunity [3]. Several types of lymphomas are known to be epidemiologically linked to autoimmune paraneoplastic manifestations with a higher risk in patients with prior autoimmunity. Autoimmunity is a high-risk factor for lymphoma manifestations, as evidenced by population studies, excluding drugs as a cause. The incidence of lymphoid malignancies is higher in people with pre-existing autoimmune diseases such as systemic lupus [58], Celiac disease [59], rheumatoid arthritis, and Sjögren’s syndrome [60], and others [60,61,62,63,64,65,66,67,68]. Moreover, in some autoimmune diseases, such as Rheumatoid arthritis (RA) and SLE, more intense autoimmune activity and/or its longer duration might signal for higher risk of lymphoma manifestation [69].

In one study, conducted on 940 lymphoma patients, 7.6% of Non-Hodgkins’ (NHL) lymphoma patients, and 8.6% of Hodgkin’s (HL) patients had a history of autoimmune disease such as skin diseases, thyroiditis, polymyositis, scleroderma, RA, vasculitis, autoimmune hepatitis, autoimmune hemolytic anemia (AIHA), and SLE [70].

Several potential mechanisms have been proposed by which autoimmunity could be associated with the risk of lymphoma development. The earliest theories pointed to the similar proliferative mechanisms of lymphocytes causing autoimmunity and hematologic malignancies [71]. Both disease types are the result of multistep processes that eliminate checkpoints that prevent autoimmune cells from growing out of control. Both inherited and somatic mutations of the genes implicated in these pathways are believed to play a role [3]. These mutations dysregulate apoptosis, enhance lymphoid hyperplasia, thus inducing both the autoimmune and malignant lymphoproliferative processes. For example, autoimmune disorders and lymphomas in mice and humans that are linked to somatic and germline *FAS* mutations [72]. Moreover, somatic *FAS* mutations identified in patients of non-Hodgkin’s lymphoma are mainly among cases with a prior history of autoimmunity [35]. Another example demonstrating overlap in the pathogenicity of autoimmunity and lymphoma is celiac disease [3], in which the inflammation remits when gluten consumption is avoided. However, in a subset of patients, the disease progress into a gluten-independent gut disorder (refractory sprue) [59]. This disease progression is usually accompanied by an oligoclonal expansion of intraepithelial T cells leading to T-cell lymphoma, suggesting that refractory sprue is associated with abnormal clonal intraepithelial lymphocytes linking celiac and T-cell lymphoma [59].

### 3.6. Somatic Mutation in Autoimmune Diseases

The first identified non-malignant autoimmune disease caused by a somatic mutation was in children with symptoms resembling lymphoproliferative syndrome (ALPS, also known as Canale-Smith syndrome) but lacked the inherited germinal mutation in the *FAS* gene (Table 1) [34]. The cause of this was found to be somatic *FAS* mutations in hematopoietic precursor stem cells, causing symptoms resembling ALPS, an accumulation of double-negative T cells, and hypergammaglobulinemia [34]. The necessity for the mutation to exist in both T- and B-cells for the condition to develop was established in mouse model experiments, suggesting that the mutation must arise early in lymphocyte development. However, sporadic autoimmunity might result from a *FAS* mutation in a single B cell or T cell clone, paired with other checkpoint deficits [3].

In MS, earlier studies using the hypoxanthine guanine phosphoribosyltransferase (HPRT) assay presented results that attest to the existence of somatic mutations in autoreactive T-lymphocytes of MS patients (Table 1) [73,74]. This assay identifies the resistance of cultured cells to 6-thioguanine, caused by somatic mutations inhibition of *HPRT* gene or other related pathways [75]. Using this approach, frequency of somatic mutations in adults T-lymphocytes was estimated to be approximately 5 × 10^−6^ [75]. Mutation frequency for *HPRT* in T lymphocytes of MS patients was estimated to be greater than that in controls [74]. Another MS study reported T-lymphocytes clones deficient for HPRT were autoreactive to myelin autoantigen, unlike wild-type clones [73].

Employing a recent approach, exome-sequencing of CD4^+^ T-lymphocytes derived the cerebrospinal fluid of two MS patients while using DNA from the patients’ peripheral blood as germline reference resulted in calling thousands of variants; however, the data presented in this study could not confidently identify true somatic mutations from potential amplification artefacts (Table 1) [76].

Another study aimed to detect potential somatic mutations and determine their frequency in 16 MS patients in different cell subpopulations using next-generation sequencing of 986 immune-related genes (Table 1) [77]. Here, 60% of tested MS patients harbored non-synonymous somatic mutations with enrichment in CD8^+^ cells in genes of an autoimmunity role such as *ATM, CLIP2, IKFZF3, MAPK10,* and *STAT3* [77]. Moreover, 96% of the mutations remained detectable in a follow-up analysis ~2.3 years later [77].

In RA, using a custom capture panel covering immune-related genes and exome sequencing of selected genes, exclusive mutations were identified in mature CD8^+^ T cells of 20% untreated newly diagnosed patients with RA affecting genes of an immune function *SLAMF6* and *IRF1* (Table 1) [78]. This finding is in line with previous findings mentioned above reporting somatic mutations in CD8^+^ T in MS.

**Figure 2:**
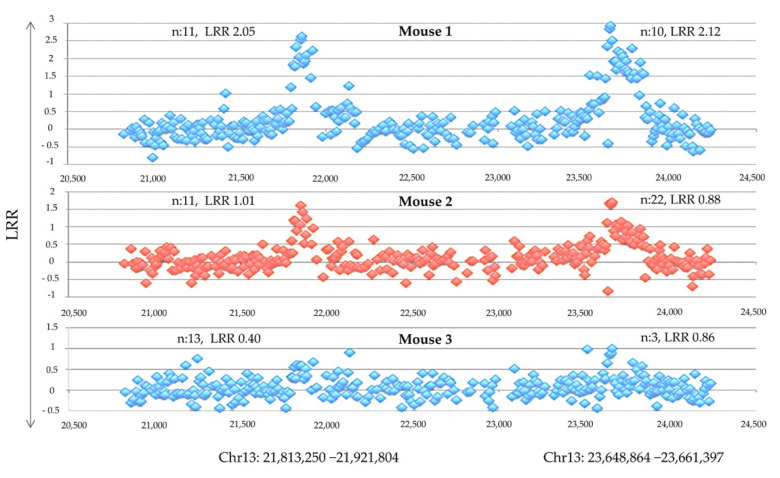
Comparative genomic hybridization (CGH) showing independent recurrent CNAs in memory cells obtained from the pancreatic lymph nodes of three different diabetic mice, spanning two histone families’ loci, collectively referred to as histone H1, H2, and H3, separated by a normal copy-number stretch encoding other genes (*n*: number of affected probes, DLRSpread values: Mouse 1: 0.28, Mouse 2: 0.23, and Mouse3: 0.19).

In a recent study by us, we aimed to test the somatic mutations hypothesis in T1D [36]. T1D is caused by both inherited susceptibility [79,80] and environmental triggers [81], however, these alone do not explain all observed in the disease. The concordance for the diseases in monozygotic twins is only 65%, with an age of onset that may differ by several decades [82]. Similarly, in the inbred nonobese diabetic (NOD) mouse model, not all mice develop the disease. The incidence in females is less than 100%, and in males is <50%, despite genetic identity and standardized environment [83]. These observations point to stochastic determinants, and one plausible such determinant is somatic mutations in the expanding antigen-specific autoreactive T cell lineages. In this study, we employed comparative genomic hybridization (CGH) on DNA from memory CD4^+^ T cells obtained from the pancreatic lymph nodes of 25 diabetic NOD mice (Table 1). Using this approach, and with additional validation via multiplex ligation-dependent probe amplification (MLPA), we identified lymphocytes-exclusive, mosaic somatic, copy-number aberrations (CNAs) with non-random involvement of the same gene(s) across different mice (e.g., *Ilf3* and *Dgka*) with autoimmunity or a proliferation-control association [36]. Two mice had a very unstable genome and numerous recurrent CNAs. CGH analysis of the memory lymphocytes genomes of these mice revealed recurrent CNAs; a third mouse of copy gains spanning the histone loci of highly homologous genes encoding nuclear histone proteins, which could explain the instability of their genomes (Figure 2) [36].

Moreover, lymphocytes expanding during normal host defense as a response to a *Leishmania major* parasite infection harbored somatic mutations that were fewer and significantly smaller than those in autoreactive cells. Here, TCR clonality analysis suggests these mutations to have a pre-thymic occurrence [36].

The studies mentioned above lay the groundwork for forthcoming studies aiming to comprehend the contribution of somatic mutations in autoimmunity and other non-malignant disorders. They unravel persistent somatic mutations, many of which were in immune-function genes or genes associated with cell proliferation. However, due to the lack of functional assays, it is neither clear whether these mutations play a role in autoimmune pathogenicity nor the exact effects these mutations have on the cell phenotype, function, and disease causation. Nevertheless, these studies demonstrate that somatic mutations in disease-causing cells are, perhaps, more than just indices of previous mitoses after the vigorous antigen-driven proliferation, making their potential role as critical players in autoimmunity an attractive, hitherto little-studied research target that warrants further investigation.

## 4. Practical Implications and Potential Benefits

There are several practical implications for the somatic mutation hypothesis in autoimmune diseases, and these stem from two properties of these mutations that make them exceptionally attractive, in the context of precision diagnostics and therapeutics. First, somatic mutations are more likely to have more severe functional effects than germinal mutations, since they do not undergo purifying selection for the fitness of the whole organism [36]. Second, somatic mutations exist in pathogenic immune cells, making them promising biomarkers and therapeutic targets, without interfering with the fitness of other lymphocytes or other cells in general. This is similar to the previously mentioned treatment for chronic myeloid leukemia, Gleevec [3,23]. Moreover, identifying driver mutations (inherited or somatic) from the proliferation accumulating passenger mutations will allow for a better understanding of the cause of various autoimmune diseases and will facilitate therapeutic advances by identifying drug targets. In line with cancer, immune cells harboring these mutations could be hypersensitive to certain drugs, in contrast to their normal counterparts and, therefore, can be selectively targeted using strategies similar to those employed in cancer [3]. For instance, some cancer drugs, such as Rituximab (first developed for the treatment of non-Hodgkin lymphoma), are efficient against various autoimmune diseases (they suppresses autoimmunity to a more considerable extent than immunity against infection) [84,85,86]. Finally, defining the mutational profile of individual patients might promote precision medicine in selecting the most appropriate treatment for each patient.

## 5. Future Research Prospects

The role of somatic mutations in the pathogenicity of non-malignant diseases, in general (and in autoimmunity, in particular), is just beginning to be acknowledged. To solidify the somatic mutations hypothesis in the pathogenicity of autoimmunity and other non-malignant diseases, further studies with high-throughput experimental setups, accounting for the vast complexity of the question, will be needed. To further understand the role of somatic mutations in autoimmunity, several questions need to be addressed; these include continuing the efforts in evaluating the full spectrum of somatic mutations (point or copy-number) in different autoimmune diseases and their frequency among patients and healthy individuals over their lifetime. More studies will be needed to identify potential mutations in various autoimmune diseases. Moreover, functional studies and the physiological impacts of these mutations require investigation to determining what cell populations are preferentially harboring these mutations, in various autoimmune diseases, and what their allelic fraction is within cell populations. Determining potential driver mutations and whether there are any mutational hotspots is also necessary.

The scientific community began to address the question of somatic mutations in non-malignant diseases and their potential role in the pathogenicity of autoimmune diseases. Extensive efforts are needed to provide more evidence and to decode the role of the somatic mutation in non-malignant diseases, autoimmunity, health, and disease.

## Figures and Tables

**Table 1:** Technical approaches for detecting somatic mutations in autoimmunity.

Technical Method	Data	Autoimmune Disease	Description	References
Sequencing/candidate gene approach	Single-nucleotide variant	Autoimmune lymphoproliferative syndrome	DNA extracted from phytohemagglutinin-activated lymphocytes or purified double-negative T cells was amplified with oligonucleotides spanning the nine *FAS* exons and sequenced	[34]
Hypoxanthine guanine phosphoribosyltransferase (HPRT) assay	Mutant frequency values	Multiple sclerosis	Identifies the resistance of cultured T cells to 6-thioguanine, caused by somatic mutations inhibition of *HPRT* gene or other related pathways	[73,74,75]
Exome-sequencing	Single-nucleotide variant and their frequency values	Multiple sclerosis	Exome-sequence DNA of CD4^+^ lymphocytes isolated from patients’ cerebrospinal fluid along with sequencing DNA from peripheral blood to serve as germline reference	[76]
Next-generation HiSeq-DNA sequencing using a custom designed gene panel consisting of 986 genes related to immunity and cancer	Single-nucleotide variant	Multiple sclerosis, Myasthenia gravis, and Narcolepsy	Somatic mutations were called using next-generation HiSeq-DNA sequencing targeting 986 immune-related genes and by comparing the sequence data of immune cell subpopulations to other subpopulations of the same patient	[77]
Custom deep-sequencing panel of immune-related genes and exome and deep amplicon sequencing	Single-nucleotide variant	Rheumatoid arthritis	Custom deep-sequencing panel of 986 immune-related genes of CD8^+^ T cells of untreated newly diagnosed patients with RA	[78]
Comparative genomic hybridization (CGH) assay and validation via multiplex ligation-dependent probe amplification (MLPA)	Copy-number aberrations	Type 1 diabetes	CGH on DNA from memory CD4^+^ T cells obtained from the pancreatic lymph nodes of diabetic NOD mice and using DNA from a tail-clip sample to serve as germline reference	[36]

## Data Availability

Not applicable.
